# Novel functionalized monomers based on kojic acid: snythesis, characterization, polymerization and evalution of antimicrobial activity

**DOI:** 10.1080/15685551.2016.1259832

**Published:** 2016-11-21

**Authors:** Mahnaz Saraei, Gholamreza Zarrini, Moshgan Esmati, Leila Ahmadzadeh

**Affiliations:** ^a^ Department of Chemistry, Payame Noor University, Tehran, Iran; ^b^ Faculty of Natural Sciences, Department of Biology, University of Tabriz, Tabriz, Iran

**Keywords:** Kojic acid, acrylate monomers, 1,2,3-Triazole, free radical polymerization, thermal properties, antimicrobial activity

## Abstract

Two novel acrylate monomers, [5-(benzyloxy)-4-oxo-4*H*-pyran-2-yl]methyl acrylate and {1-[(5-(benzyloxy)-4-oxo-4*H*-pyran-2-yl)methyl]-1,2,3-triazol-4-yl}methyl acrylate were synthesized by the reaction of 5-benzyloxy-2-(hydroxymethyl)-4*H*-pyran-4-one and 5-(benzyloxy)-2-{[4-(hydroxymethyl)-1,2,3-triazol-1-yl]methyl}-4*H*-pyran-4-one with acryloyl chloride in the presence of triethylamine, respectively. These monomers were polymerized using 2,2^′^-azobisisobutyronitrile (AIBN) as the initiator in *N*,*N*-dimethylformamide:14-dioxane (10:1) solution. The thermal behavior of the polymers was investigated by thermogravimetric analysis (TGA) and differential scanning calorimetry (DSC). The synthesized compounds were evaluated for their antibacterial and antifungal activites aganist bacteria and fungi using the disk diffusion method. The results indicated that some of these compounds demonstrated moderate to good antibacterial and antifungal activities*.*

## 1. Introduction

Kojic acid, 5-hydroxy-2-hydroxymethyl-4*H*-pyran-4-one, is an organic acid which produced by many species of fungi and bacteria, such as *Aspergillus*, *Acetobacter* and *Penicillium* in an aerobic process from a wide range of carbon sources.[1–3] Kojic acid and its derivatives have attracted much attention because various biological activities such as antioxidant,[4] anticancer,[5] antidiabetic,[6] antimicrobial, antiviral,[7] antiproliferative,[8] anti-inflammatory,[9] antispeck,[10] pesticide and insecticide.[11] It is a bidentate metal chelator and is considered as a potent iron chelator.[12,13] In addition, kojic acid and its derivatives are used in the food industry as an additive to prevent the browning of food materials and as a skin-lightening agent in cosmetic products, due to the its tyrosinase inhibitory activity.[14,15]

Functional polymers have become increasingly important due to their various applications such as polymeric reagents and crosslinking resins, etc.[16,17] These polymers can be prepared either by synthesizing new functional monomers and their subsequent polymerization or by converting functional groups on the polymer into the desired functional groups.[18–21] Functional groups give the polymer structure of special characters substantially different from the inherent properties of the basic polymer.[22] Functional polymers such as acrylates have attracted much attention because their wide range of applications in adhesives, coating, paper and textiles industries.[23–26]

There are many reports on the synthesis of acrylate monomers and their polymers.[27–31] As the best of our knowledge, the synthesis of monomers containing kojic acid moiety has not been reported.[32] Herein, we wish to report the synthesis and characterization of two novel acrylate monomers containing kojic aicd moiety and their polymers. The thermal stability of the polymers was studied by differential scanning calorimeter (DSC) and thermogravimetric analysis (TGA). Also, the synthesized compounds were investigated for their antimicrobial activities against Gram-positive and Gram-negative bacteria and fungi.

## 2. Experimental

### 2.1. Material and methods

All reagents were purchased from Merck and Fluka companies and were used without further purification. Solvents were dried and distilled prior to use. All reactions were followed by thin layer chromatography (TLC) on silica gel 60 HF_254_, with detection by UV light. Crude products were purified by preparative layer chromatography (PLC; Merck, silica gel 60 F_254_, CAMAG, Switzerland) using *n*-hexane: acetone (2:1) as eluent. Melting points were determined on an Electrothermal Barnstead 9200 apparatus (Barnstead, UK). FT-IR spectra were obtained using KBr pellets on a tensor 27-Bruker spectrometer (Shimadzu, Japan). ^1^H NMR and ^13^C NMR spectra were recorded with a FT-NMR-Bruker spectrometer (Germany), at 400 and 100 MHz, respectively, in CDCl_3_ and DMSO-*d*_*6*_. Mass spectra were recorded on direct insert probe Agilent Technologies 5975c with triple axis detector. Elemental analyses were carried out on Perkin-Elmer CHNS-O Analyzer, Model 2400 Series II and were found to agree favorably with the calculated values. The thermogravimetric analysis (TGA) of the polymers was performed under nitrogen atmosphere at a heating rate of 20 °C min^−1^ using a Linseis STA PT1000 thermal analyzer. The glass transition temperature (*T*_g_) of the polymers was measured using a differential scanning calorimetry (DSC), TGA/SDTA 851, under nitrogen atmosphere at a heating rate of 10 °C min^−1^.

### 2.2. Synthesis of [5-(benzyloxy)-4-oxo-4H-pyran-2-yl]methyl acrylate (2)

*Method 1*: To a suspension of 5-benzyloxy-2-(hydroxymethyl)-4*H*-pyran-4-one **1b** (0.23 g, 1 mmol) in 10 mL dry THF under nitrogen atmosphere was added Et_3_N (0.51 g, 5 mmol), and the reaction mixture was cooled to 0 °C using ice bath. Then, acryloyl chloride (0.45 g, 5 mmol) in 2 mL THF was added dropwise to the reaction mixture, and the resulting mixture was stirred at 0 °C for 2 h. After completion of the reaction, monitored by TLC, the solvent was removed under reduced pressure and the crude product was purified by PLC on silica gel with *n*-hexane: acetone (2:1) as an eluent. 0.13 g (46%); Cream solid; mp: 95–98 °C; FT-IR (KBr): ῡ = 3078, 2920, 1733 (ester C=O), 1650 (pyrone C=O), 1625, 1517, 1506, 1450, 1406, 1269, 1193, 937, 736 cm^−1^; ^1^H NMR (400 MHz, CDCl_3_): *δ* = 4.97 (s, 2H, –CH_2_O–), 5.08 (s, 2H, –CH_2_OCO–), 5.96 (dd, 1H, *J* = 0.8 and 10.4 Hz), 6.17 (dd, 1H, *J* = 10.4 and 17.2 Hz), 6.47 (s, 1H, pyrone-H), 6.51 (dd, 1H, *J* = 0.8 and 17.2 Hz), 7.33–7.41 (m, 5H, Ar-H), 7.55 (s, 1H, pyrone-H) ppm; ^13^C NMR (100 MHz, CDCl_3_): *δ* = 61.1, 71.9, 114.1, 127.1, 127.8, 128.5, 128.7, 132.8, 135.6, 141.4, 147.3, 161.4, 164.9, 174.3 ppm; Ms: m/z (%) = 286 (M^+^, 34), 91 (100). Anal. Calcd. for C_16_H_14_O_5_: C, 67.13; H, 4.93. Found: C, 67.30; H, 4.83.

*Method 2*: To a solution of compound **1b** (0.23 g, 1 mmol) and acrylic acid (0.14 g, 2 mmol) in 10 mL dry CH_2_Cl_2_ under nitrogen atmosphere was added a solution of *N,N’*-dicyclohexylcarbodiimide (DCC) (0.23 g, 1.1 mmol) in 2 mL CH_2_Cl_2._ Then, a solution of 4-(*N,N*-dimethylamino)pyridine (DMAP) (0.12 g, 1 mmol) in 1 mL CH_2_Cl_2_ was added to above solution. The reaction mixture was heated at 60–70 °C for 72 h. After completion of the reaction, monitored by TLC, the precipitated dicyclohexyl urea was filtered off and the solvent was removed under reduced pressure. The residue was purified by PLC on silica gel with *n*-hexane: acetone (2:1) as an eluent to give the pure monomer in 67% yield.

### 2.3. Synthesis of {1-[(5-(benzyloxy)-4-oxo-4H-pyran-2-yl)methyl]-1,2,3-triazol-4-yl}methyl acrylate (6)

To a suspension of 5-(benzyloxy)-2-{[4-(hydroxymethyl)-1,2,3-triazol-1-yl]methyl}-4*H*-pyran-4-one **5** (0.1 g, 0.3 mmol) in 10 mL dry THF was added Et_3_N (0.15 g, 1.5 mmol) under nitrogen atmosphere and the reaction mixture was cooled to 0 °C using ice bath. Then, acryloyl chloride (0.14 g, 1.5 mmol) in 2 mL THF was added dropwise, and the reaction mixture was stirred at 0 °C for 2 h. After completion of the reaction, monitored by TLC, the solvent was removed under reduced pressure and the crude product was purified by PLC on silica gel with *n*-hexane: acetone (2:1) as an eluent. 0.048 g (41%); Orange oil; FT-IR (KBr): ῡ = 3091, 3002, 2960, 1724 (ester C=O), 1652 (pyrone C=O), 1438, 1413, 1326, 1269, 1207, 1049, 979, 752 cm^−1^; ^1^H NMR (400 MHz, CDCl_3_): *δ* = 5.00 (s, 2H, –CH_2_N–), 5.28 (s, 2H, –CH_2_O–), 5.35 (s, 2H, –CH_2_OCO–), 5.85 (dd, 1H, *J* = 1.6 and 10.4 Hz), 6.11 (dd, 1H, *J* = 10.4 and 17.2 Hz), 6.30 (s, 1H, pyrone-H), 6.42 (dd, 1H, *J* = 1.2 and 17.2 Hz), 7.30–7.35 (m, 5H, Ar-H), 7.52 (s, 1H, triazole-H), 7.78 (s, 1H, pyrone-H) ppm; ^13^C NMR (100 MHz, CDCl_3_): *δ* = 50.6, 57.4, 71.7, 115.0, 124.7, 127.8, 128.0, 128.6, 128.8, 131.8, 135.2, 141.1, 143.6, 147.4, 159.3, 165.9, 173.9 (pyrone C=O).

### 2.4. Radical polymerization of [5-(benzyloxy)-4-oxo-4H-pyran-2-yl]methyl acrylate

To a solution of acrylate monomer **2** (0.5 g, 1.75 mmol) in 15 mL DMF:14-dioxane (10:1) under nitrogen atmosphere was added AIBN (0.02 g, 4% of the monomer concentration), and the reaction mixture was heated at 90 °C for 72 h. The resulting polymer was precipitated by adding the reaction mixture into methanol. The polymer was filtered off and dried in vacuum. Brown solid, FT-IR (KBr): ῡ = 3078, 2952, 1743 (ester C=O), 1650 (pyrone C=O), 1515, 1382, 1263, 1205, 1157, 1016, 884, 742 cm^−1^; ^1^H NMR (400 MHz, CDCl_3_): *δ* = 1.54–2.03 (br, 2H, –CH_2_– backbone), 2.40–2.75 (br, 1H, –CH– backbone), 2.88 (s, 2H, –CH_2_O–), 2.96 (s, 2H, –CH_2_OCO–), 6.40 (s, 1H, pyrone-H), 7.14–7.36 (m, 5H, Ar-H), 7.56 (s, 1H, pyrone-H) ppm.

### 2.5. Radical polymerization of {1-[(5-(benzyloxy)-4-oxo-4H-pyran-2-yl)methyl]-1,2,3-triazol-4-yl}methyl acrylate

To a solution of acrylate monomer **6** (0.5 g, 1.36 mmol) in 20 mL DMF:14-dioxane (10:1) under nitrogen atmosphere was added AIBN (0.04 g, 8% of the monomer concentration), and the reaction mixture was heated at 90 °C for 24 h. The resulting polymer was precipitated by adding the reaction mixture into methanol. The polymer was purified by repeated reprecipitation by methanol from a solution of the polymer in DMF. The polymer was filtered off and dried in vacuum. Brown solid, FT-IR (KBr): ῡ = 3050, 2943, 1754 (ester C=O), 1650 (pyrone C=O), 1521, 1479, 1390, 1267, 1174, 1035, 806 cm^−1^; ^1^H NMR (400 MHz, DMSO-*d*_*6*_): *δ* = 1.10 (br, 2H, –CH_2_– backbone), 1.29 (br, 1H, –CH– backbone), 2.54 (s, 2H, –CH_2_ N–), 2.73 (s, 2H, –CH_2_O–), 2.87 (s, 2H, –CH_2_OCO–), 6.41 (s, 1H, pyrone-H), 7.30–7.37 (m, 5H, Ar-H), 7.95 (s, 1H, triazole-H) 8.18 (s, 1H, pyrone-H) ppm.

### 2.6. Antimicrobial activity assay

The antibacterial and antifungal activities of the synthesized compounds **2** and **5–8** were determined against bacteria, *Staphylococcus aureus, Bacillus subtilis, Escherichia coli, Salmonella typhi* and a fungus, *Candida kefyr* by the standard disc diffusion method. Muller-Hinton agar (MHA) (oxoid) and sabouraued dextrose agar (SDA) were used for preparation of the media for bacteria and fungi strains, respectively. The filter paper discs (6.4 mm in diameter) were individually impregnated with 10 μL of stock solution of the extracts (2 mg/disc) and then placed onto the agar plates which had previously been inoculated with the tested microorganisms. The plates were inoculated with bacteria incubated at 37 °C for 18–24 h and at 30 °C for 48 h for fungal strain. The diameters of inhibition zones were measured in millimeters. All the tests were performed in duplicate. Gentamicin (30 μg) and Amphotricin B (50 μg) served as positive control.

## 3. Results and discussion

**Scheme 1.**
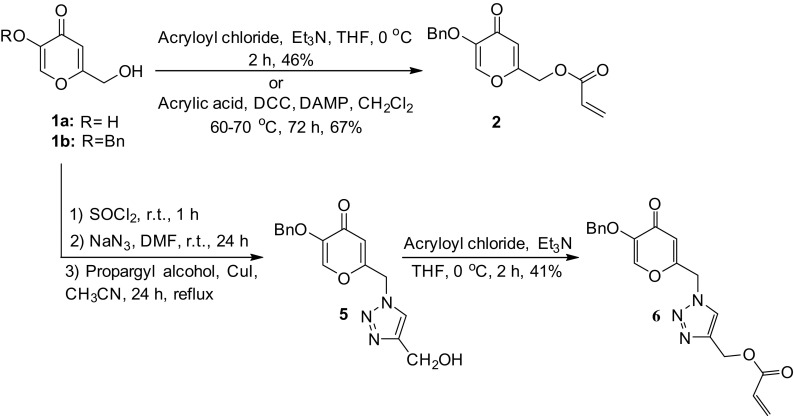
Synthesis of acrylate monomers **2** and **6** **.**

In the present work, two novel acrylate monomers, [5-(benzyloxy)-4-oxo-4*H*-pyran-2-yl]methyl acrylate **2** and {1-[(5-(benzyloxy)-4-oxo-4*H*-pyran-2-yl)methyl]-1,2,3-triazol-4-yl}methyl acrylate **6** were synthesized by the esterification of 5-benzyloxy-2-(hydroxymethyl)-4*H*-pyran-4-one **1b** and 5-(benzyloxy)-2-{[4-(hydroxymethyl)-1,2,3-triazol-1-yl]methyl}-4*H*-pyran-4-one **5** with acryloyl chloride in the presence of triethylamine in 46% and 41% yields, respectively. Also, acrylate monomer **2** was synthesized by the reaction of compound **1b** with acrylic acid in the presence of *N,N’*-dicyclohexylcarbodiimide (DCC) and 4-(*N,N*-dimethylamino)pyridine (DMAP) in 67% yield (Scheme 1). For this purpose, compound **1b** was prepared by the reaction of kojic acid **1a** with benzyl chloride according to the literature procedure.[33] 5-Benzyloxy-2-(chloromethyl)-4*H*-pyran-4-one **3** was prepared by the reaction of compound **1b** with thionyl chloride at room temperature, which was converted to 2-(azidomethyl)-5-benzyloxy-4*H*-pyran-4-one **4** using sodium azide in dry DMF at room temperature.[34,35] 1,4-Disubstituted 1,2,3-triazole **5** was prepared by the reaction of azide compound **4** with propargyl alcohol in the presence of CuI as catalyst in dry acetonitrile under reflux conditions for 24 h.

The structures of the acrylate monomers were established on the basis of FT-IR, ^1^H NMR, ^13^C NMR, MS and elemental analysis. In the FT-IR spectra of the monomers **2** and **6**, the strong absorption bands at 1730 and 1724 cm^−1^ are assigned to the stretching vibration of the ester carbonyl groups, respectively.

**Figure 1.**
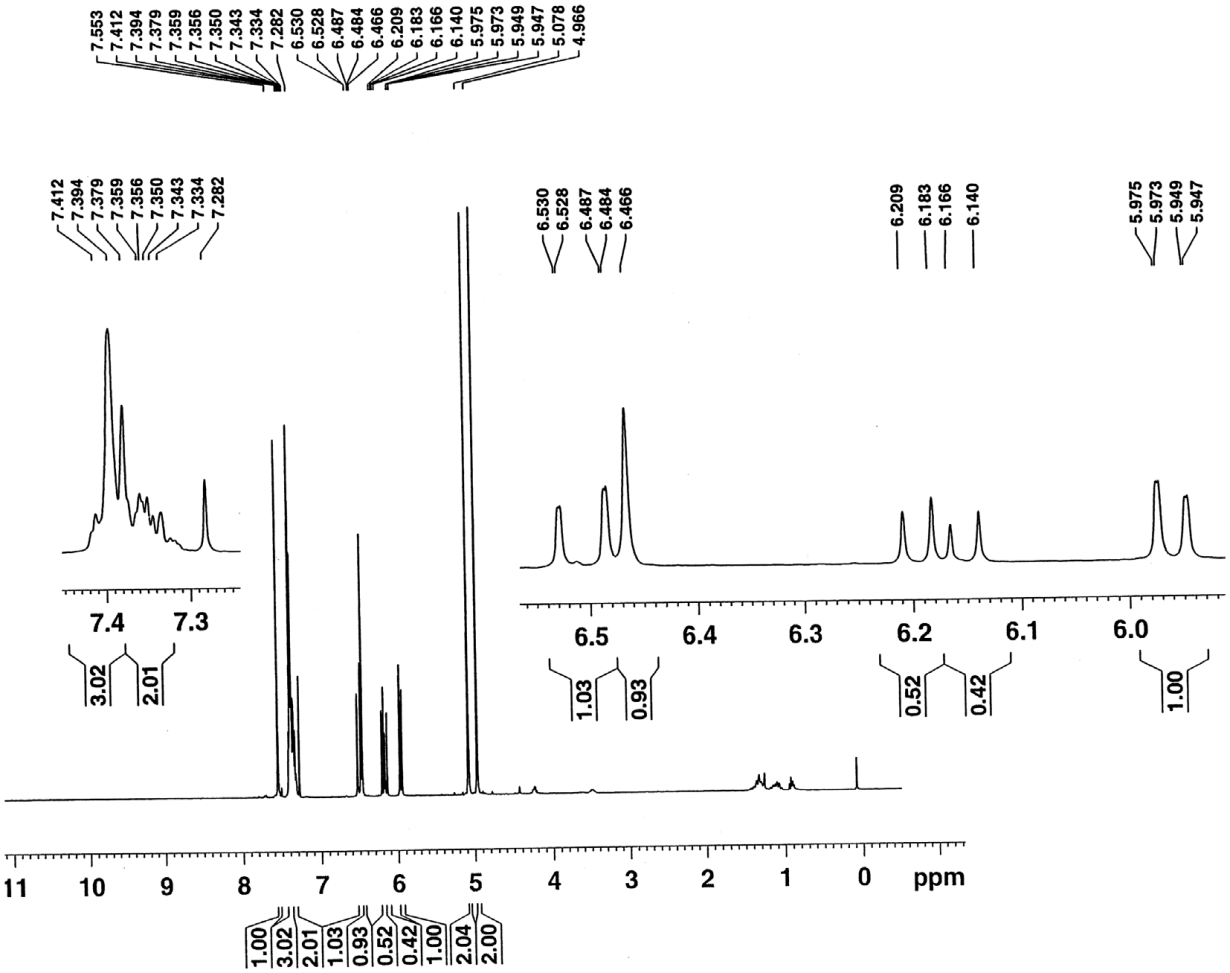
^1^H NMR spectrum of the monomer **2** in CDCl_3__._

**Figure 2.**
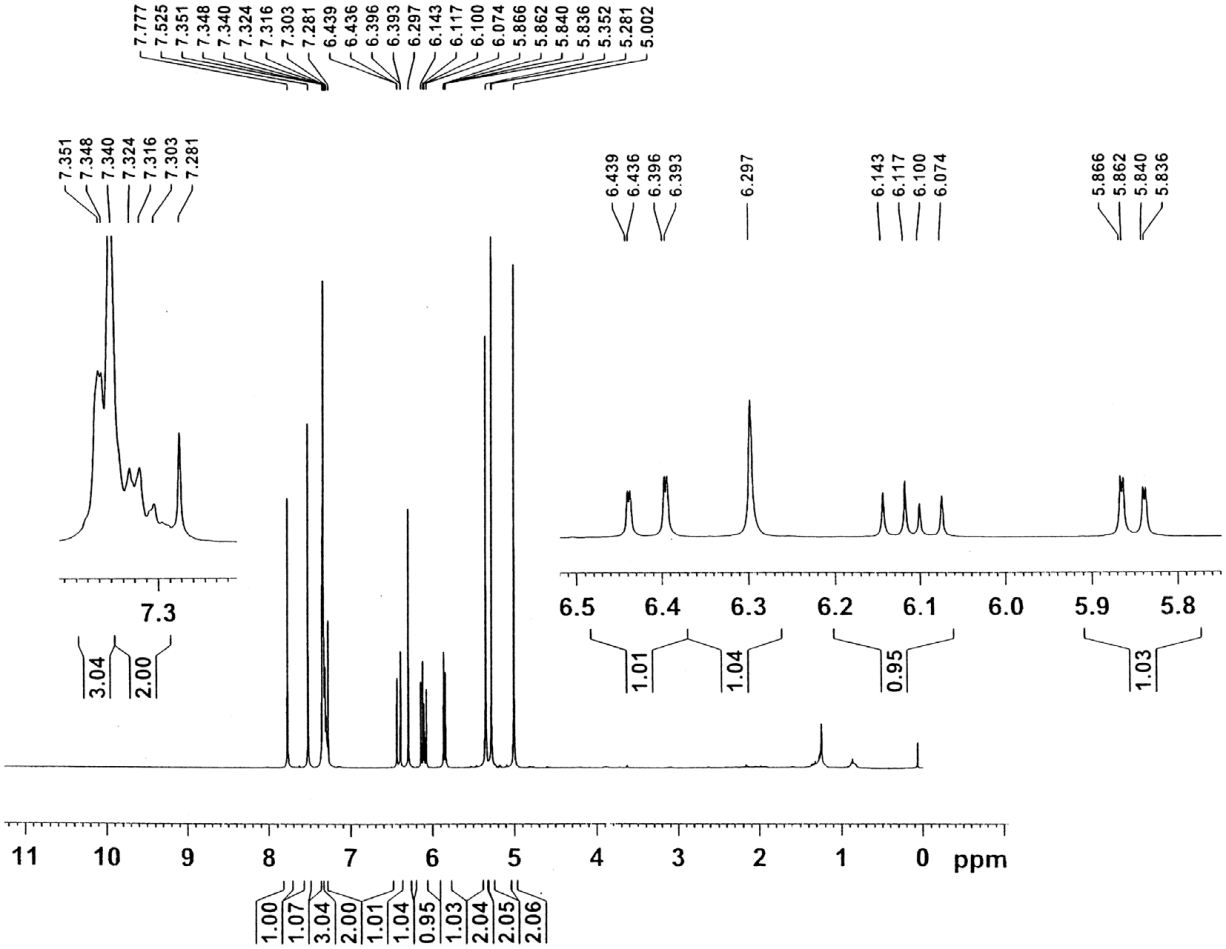
^1^H NMR spectrum of the monomer **6** in CDCl_3__._

Figures 1 and 2 show the ^1^H NMR spectra of the monomers **2** and **6**. In the ^1^H NMR spectrum of monomer **2**, signals at 4.97 and 5.08 ppm are assigned to the –CH_2_O– and –CH_2_OCO– protons, respectively. The signals corresponding to the vinyl protons (CH_2_=CH–) from acrylate unit are assigned at 5.96, 6.17 and 6.51 ppm, signals at 6.47 and 7.55 ppm are assigned to the pyrone protons, signals due to the aromatic protons are assigned at 7.33–7.41 ppm. In the ^1^H NMR spectrum of monomer **6**, signals at 5.00 and 5.28 ppm are assigned to the –CH_2_N– and –CH_2_O– protons, respectively. A signal at 5.35 ppm is assigned to the –CH_2_OCO– protons, signals corresponding to the vinyl protons (CH_2_=CH–) from acrylate unit are assigned at 5.85, 6.11 and 6.42 ppm, signals at 6.30 and 7.78 ppm are assigned to the pyrone protons, signals due to the aromatic protons are assigned at 7.30–7.35 ppm. A signal at 7.52 ppm is assigned to the triazole proton. The results above confirmed that the monomers **2** and **6** were successfully synthesized.

**Figure 3.**
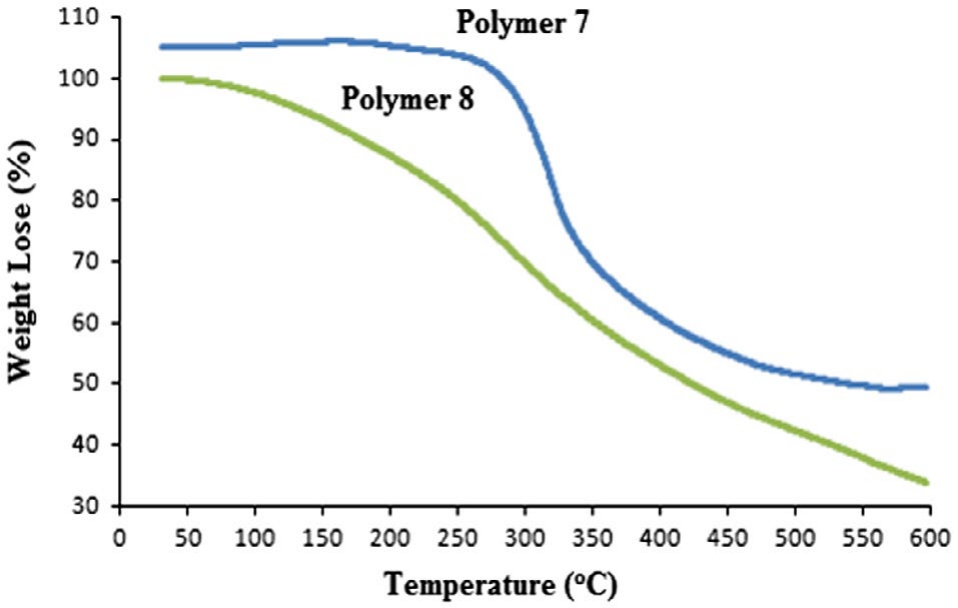
TGA thermograms of the polymers **7** and **8**.

**Scheme 2.**
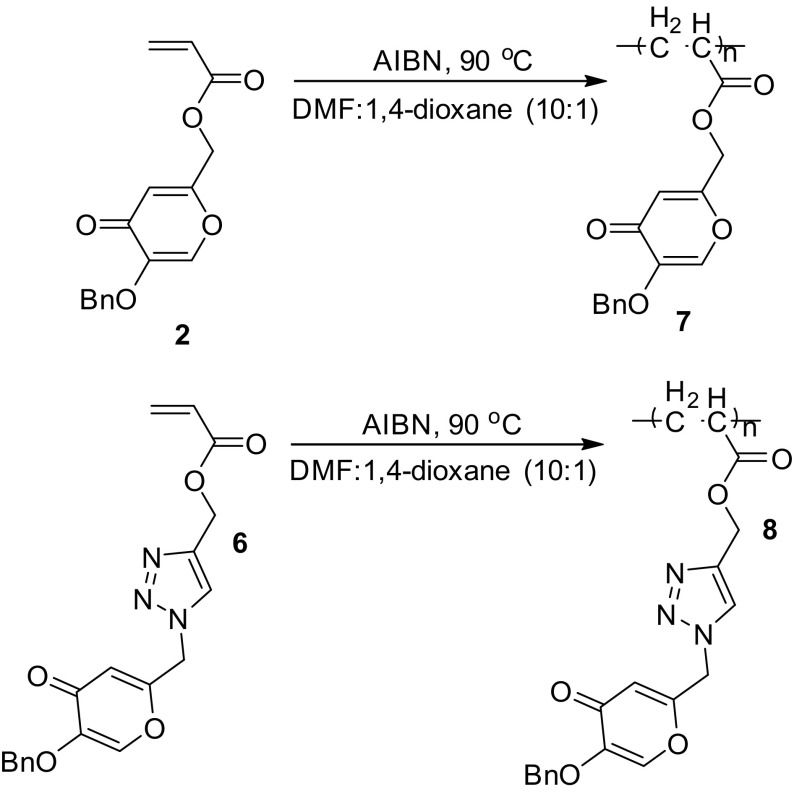
Synthesis of polymers **7** and **8**.

As shown in Scheme 2, the polymerization of the monomers **2** and **6** was carried out at 90 °C in DMF:14-dioxane (10:1) using AIBN as an initiator under nitrogen atmosphere. The obtained polymers were characterized by FT-IR, ^1^H NMR, DSC, and TGA methods. The FT-IR spectra of the polymers **7** and **8** showed the absorption bands at 1743 and 1754 cm^−1^ corresponding to the ester carbonyl groups stretching vibration, respectively.

In the ^1^H NMR spectrum of the polymer **7**, the broad peaks at 1.54–2.03 and 2.40–2.75 ppm are assigned to –CH_2_– and –CH– protons in the polymer chain, respectively. The signals at 2.88 and 2.96 ppm are assigned to the –CH_2_O– and –CH_2_OCO– protons, respectively, signals at 7.14–7.36 ppm are assigned to the aromatic protons, signals at 6.40 and 7.56 ppm are assigned to pyrone protons. In the ^1^H NMR spectrum of the polymer **8**, the broad signals at 1.10 and 1.29 ppm are assigned to –CH_2_– and –CH– protons in the polymer chian, respectively. The signals at 2.54, 2.73 and 2.87 ppm are assigned to the –CH_2_N–, –CH_2_O– and –CH_2_OCO– protons, respectively, signals at 7.30–7.37 ppm are assigned to the aromatic protons, signals at 6.41 and 8.18 ppm are assigned to pyrone protons, signal at 7.95 ppm is assigned to triazole proton. In the ^1^H NMR spectra of the polymers, the peaks due to the vinyl protons of the monomers have disappeared and the chemical shifts are well consistent with the polymers structure.

The thermal behavior of the polymers was investigated by TGA and DSC. The TGA curves of the polymers **7** and **8** are shown in the Figure 3. The TGA curve of the polymer **7** clearly indicates that weight loss of the polymer occurs in two stages. A weight loss in the temperature range of 180–250 °C may be due to absorbed solvent. The second step of weight loss in the temperature range of 250–570 °C depending on the polymer structure and may be attributed to the decomposition of the polymer and the ester linkage. At temperature 600 °C,about 44% of the initial weight of the polymer is remained and this shows that the polymer has good thermal stability. The TGA curve of the polymer **8** exhibits a continuous weight loss stage in the temperature range 30–600 °C, corresponding to the polymer structure, the ester linkage and heterocyclic rings. At temperature 600 °C, about 33% of the initial weight of the polymer is remained and this shows that the polymer has good thermal stability. The glass transition temperature (*T*_g_) of the polymers was determined by DSC. The polymers **7** and **8** were indicated a single *T*_g_ at 48.24 and 61.75 °C, respectively. The polymers were soluble in *N,N*-dimethylformamide and dimethyl sulfoxide, but insoluble in chloroform, dichloromethane, tetrahydrofuran, 14-dioxane, methanol, ethanol, *n*-hexane, acetone, ethyl acetate, acetonitrile and toluene.

### 3.1. Antimicrobial activity

The synthesized compounds **2** and **5–8** were evaluated for their antibacterial activity against two gram-positive bacteria *Staphylococcus aureus, Escherichia coli* and two gram-negative bacteria *Escherichia coli* and *Salmonella typhi*. They were also evaluated for their *in vitro* antifungal activity against *Candida kefyr*. The compounds **2**, **5** and **6** were showed good or significant antifungal activity with inhibition zones about (9.6–17 mm) as compared to standard drug Amphotricin B. These compounds were indicated moderate antibacterial activity against the gram-positive bacteria with inhibition zones about (8–9 mm) and a weak antibacterial activity against the gram-negative bacteria as compared to standard drug Gentamicin. The polymers **7** and **8** were indicated lower activity or no activity on the gram-positive and the gram-negative bacteria and fungi.

## 4. Conclusion

In this study, we have synthesized two novel acrylate monomers containing kojic acid moiety by the reaction of 5-benzyloxy-2-(hydroxymethyl)-4*H*-pyran-4-one and 5-(benzyloxy)-2-{[4-(hydroxymethyl)-1,2,3-triazol-1-yl]methyl}-4*H*-pyran-4-one with acryloyl chloride or acrylic acid. These monomers were polymerized using 2,2^′^-azobisisobutyronitrile (AIBN) as the initiator. The thermal stability of the polymers was studied by TGA analysis and showed that the resulting polymers have good thermal stability, so that at temperature 600 °C, the polymers **7** and **8** are retained about 44% and 33% of theirs original weight. All the title compounds have been investigated for their antimicrobial activities. The results of antimicrobial assay indicated that some of these compounds exhibited considerable antimicrobial activity.

## Disclosure statement

No potential conflict of interest was reported by the authors.
